# Correspondence

**Published:** 1868-12-15

**Authors:** J. M. Harnett

**Affiliations:** Shelbyville, Ill.


### *CORRESPONDENCE.*

SHELBYVILLE, ILL., *November* 30*th*, 1868.

*To the Editors* CHICAGO MEDICAL JOURNAL:

If you think the following will be of sufficient interest to your readers, please insert.

### TOBACCO AS A CAUSE OF INDIGESTION AND HYPOCHONDRIA.

For some twenty-seven years I was an inveterate chewer of tobacco, and during nearly all this time I suffered severely from indigestion. I could eat scarcely any thing that did not give me severe pain, with a great thirst for water. I became almost a skeleton, and suffered from lowness of spirits. I passed large quantities of urine, having frequently to get up three and four times during the night to evacuate my bladder, passing from two to three quarts of water. The consequence was, instead of being refreshed I would rise from my bed wearied and exhausted. I dieted, and took various kinds of medicine, all with only very temporary relief. At times I was scarcely able to attend to business; life was really more burthensome than otherwise. I was convinced that tobacco was injuring me. I resolved to quit it. It is now nearly two years since I parted with my old friend tobacco, the result is, I have gained some twenty-five pounds in flesh, can eat almost any thing with impunity; can sleep all night, without being disturbed with my urinary organs, and, in short, feel like a new man. I would say to the craft, if there are any suffering from dyspepsia or lowness of spirits, if you use the weed, *tobacco*, quit it immediately.

Yours respectfully,

J. M. HARNETT.

